# Abdominal Wall Endometriosis: A Case Report and Literature Review of Pfannenstiel Incision Endometrioma

**DOI:** 10.7759/cureus.66223

**Published:** 2024-08-05

**Authors:** Nathan Zhang, Sedona Robrahn, Katherine R Thornburgh, Justin Moon, Muhammad K Ather, Colton P Boney, Joel A Yalowitz

**Affiliations:** 1 Medicine, Alabama College of Osteopathic Medicine, Dothan, USA; 2 Research, Alabama College of Osteopathic Medicine, Dothan, USA; 3 Radiology, Decatur Morgan Hospital, Decatur, USA

**Keywords:** ct imaging, abdominal-wall endometriosis, endometriosis excision, scar site endometriosis, tumor imaging, ob-gyn, scar endometrioma, open excision, minimally invasive interventional radiology, general radiology

## Abstract

We depict a unique case of a 39-year-old woman who presented to the emergency department with complaints of right upper quadrant pain. Work-up and a computed tomography (CT) scan revealed acute cholecystitis and the patient underwent laparoscopic cholecystectomy without complication. At this time, an incidental mass was discovered in the subcutaneous fat adjacent to the abdominal wall. The patient returned six months later with progressive, cyclic abdominal pain since her last hospital admission. Initial admission lab work was within normal limits and a urine pregnancy test was negative. Physical exam revealed tenderness around her previous cesarean section scar. Repeat CT revealed an enlarging, spiculated mass adherent to the abdominal wall. After imaging confirmation, the patient underwent complete open surgical excision for the removal of the mass. Post-surgical biopsy confirmed endometrial gland and stroma consistent with abdominal wall endometrioma. The patient was discharged with adjuvant therapy and recommended follow-up with the surgeon and her obstetrician-gynecologist. The radiological diagnosis, guidelines, and decision-making for initiating interventional treatment are discussed in this report. Our purpose in documenting this case is to present a rare diagnosis of an atypical location for an endometrioma on the abdominal wall, in a patient with prior cesarean delivery. Although this patient was treated with open excision, different interventional radiology treatments from radiofrequency ablation and focused ultrasound were discussed. In doing so, we hope to contribute to the systematic literature review on surgical excision as a treatment option for Pfannenstiel incision endometrioma.

## Introduction

Endometriosis is a disease that affects around 10-15% of reproductive-age women and is defined as functional endometrial stroma and glands located outside of the uterus [1]. Symptoms include infertility, chronic pain, dysuria, dyschezia, dysmenorrhea, and dyspareunia [1]. The most common locations of endometriosis include the pelvis, specifically the ovaries; however, it can also involve the fallopian tubes, uterosacral ligaments, or surrounding peritoneum. Less common locations include the urinary bladder, gastrointestinal tract, or soft tissues, such as the cervix, vagina, vulva, and abdominal wall [2]. 

Although there are many theories about the cause of endometriosis, the most accepted is retrograde menstruation during a menstrual period. Blood travels out of the fallopian tubes and into the pelvis, causing endometrial lesions including endometriomas. Endometriomas, known colloquially as “chocolate cysts”, are formed when ectopic endometrial tissue, glands, and stroma bleed in response to normal hormonal signaling. This hormonal bleeding may result in a hematoma that is lined by fibrous tissue, forming a cystic lesion [3]. Endometriomas are associated with more severe disease and are present in 17-44% of women with endometriosis [3]. Endometriomas can occur in many locations, most commonly in the pelvic region. However, endometriomas can appear atypically in extra-pelvic locations. They are most commonly located on the ovaries but have also been found in the bowel as well as in prior surgical incisions [3]. 

One such example of an atypical location of endometrioma is the Pfannenstiel incision scar tissue. The Pfannenstiel incision is the preferred approach for cesarean delivery and is a slightly curved incision located approximately 2-3 cm above the symphysis pubis [4]. When endometrial tissue seeding has taken place at the site of a surgical scar, it is then termed as “incisional endometriosis” [5]. While rare, the occurrence of endometriosis in a Pfannenstiel incision post-cesarean section has been reported with multiple studies indicating an infrequent incidence of no more than 1% of all patients who have undergone a cesarean section [4,5]. One proposed mechanism suggests that incisional endometriosis could be primarily due to the surgical displacement of tissue accentuated by normal physiologic changes in the post-partum period. These include the hyper-estrogen state following obstetrical delivery and the vasogenic and irritative changes from vascular growth factors, inflammatory cytokines, and weakened cellular immunity [6]. Clinical suspicion of Pfannenstiel endometrioma increases with dysmenorrhea, a cyclic, abdominopelvic pain along with an associated superficial mass. However, this diagnosis is difficult to make clinically as the symptomatology overlaps with many other common gastrointestinal and obstetric pathologies.

For the best imaging method, experts agree that initial abdominal ultrasound (AUS) is preferred with sequential computed tomography (CT), or magnetic resonance imaging (MRI) depending on the sonographic architectural findings [7,8]. While imaging is the current mainstay of guiding clinical management, definitive diagnosis and treatment occur through excisional biopsy and histological analysis, which often does not occur due to the relatively low incidence and often, asymptomatic presentation of smaller endometriomas [5,7]. In this report, we explore a case of a young woman with Pfannenstiel endometrioma to better understand the role of imaging in atypical endometrioma diagnosis and treatment.

## Case presentation

**Figure 1:**
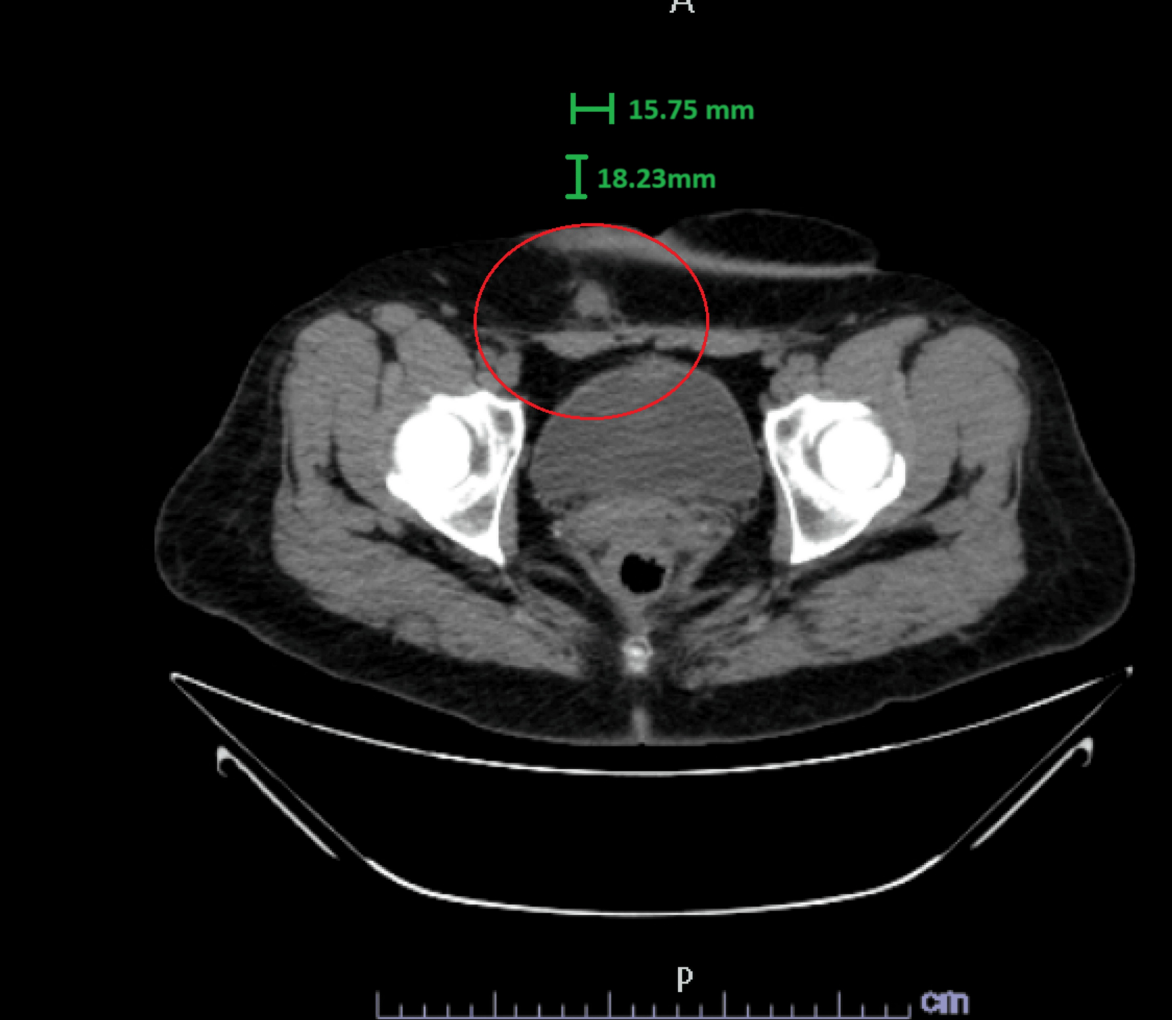
Axial CT abdomen view of the incidental 1.8 cm nodule. The nodule is well-circumscribed with homogeneous soft-tissue density and margins.

**Figure 2:**
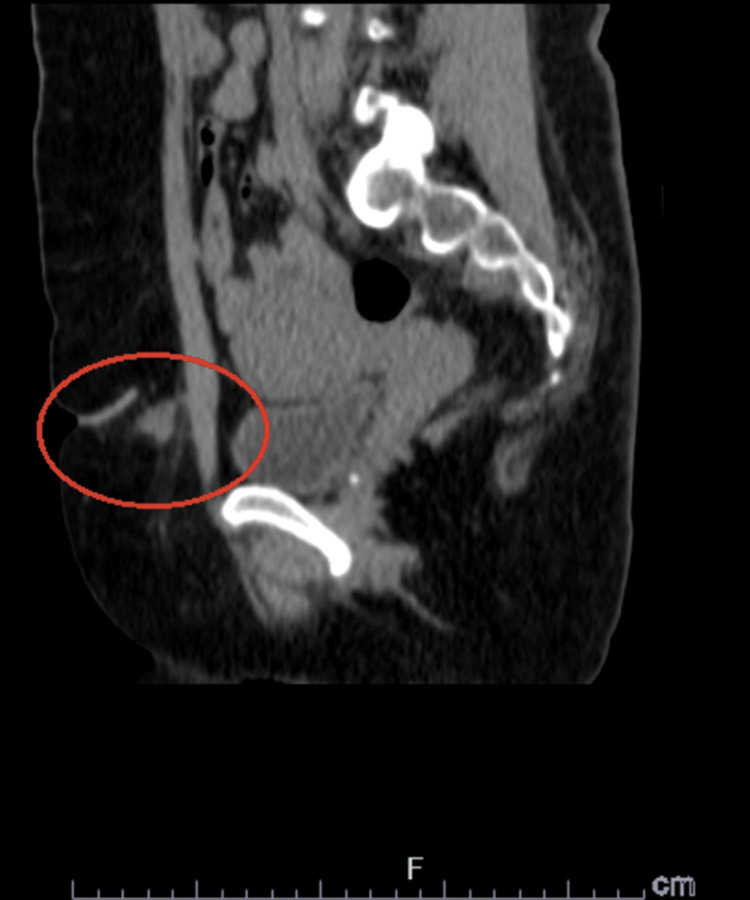
Sagittal CT abdomen view of the incidental 1.8 cm nodule. The nodule is well-circumscribed with homogeneous soft-tissue density and margins.

A 39-year-old gravida 2, para 2 female presented to the hospital with progressively worsening right upper quadrant abdominal pain since yesterday evening after dinner. She had extreme difficulty sleeping and was unable to keep down her food. She had attempted to control the pain with over-the-counter analgesics but ultimately decided to visit the Emergency Department (ED) after she developed a low-grade fever and an episode of vomiting later the next morning. She had a history of well-controlled migraine headaches, major depressive disorder, Bipolar I, and attention deficit hyperactivity disorder. She had no significant surgical history aside from one cesarean section via Pfannenstiel incision and a laparoscopic gastric bypass. She was several months postpartum and had no difficulty with postpartum care. She has no history of illicit drug use and is a non-smoker and non-drinker. Home medications included propranolol, bupropion, lamotrigine, and dextroamphetamine-amphetamine (Adderall©) for her psychiatric comorbidities. She denied any recent illness, upper respiratory symptoms, dietary changes, or sick contacts. The reports that her diet consisted primarily of processed food and her body mass index (BMI) was 34.3. The patient was later diagnosed with acute cholecystitis and the surgeon on call was able to remove the gallbladder laparoscopically with no complications. As part of the surgical workup, a computed tomography (CT) scan revealed a well-circumscribed, soft tissue mass near the inferior rectus abdominis muscle (Figures 1, 2).

Through shared decision-making, the patient agreed to defer any intervention for the nodule and to proceed with watchful waiting. This decision was made jointly due to the location and small size (<2 cm) of the nodule, as well as the lack of lower quadrant abdominal pain or mass-effect symptoms.

Five months later, the patient returned to the hospital with another episode of abdominal pain. The patient denied any significant trauma, sick contacts, or dietary changes since she was last seen in the hospital months prior. She stated the pain had been dull and progressive, and poorly localized with diffuse discomfort around the right lower quadrant. She also stated that the pain had worsened recently, and often occurred during her menses and was worried about permanent complications to her future fertility. On further history, the patient denied any menorrhagia, melena, or hematochezia and stated her menses occurred monthly and usually lasted 4-5 days with moderate flow. Initial lab work included a complete blood count (CBC), comprehensive metabolic panel (CMP), and quantitative beta-hCG to assess for ectopic pregnancy. Admission blood work and vital signs were grossly within normal limits (Table 1). 

**Table 1:** Admission lab work obtained on return hospital visit including complete blood count, comprehensive metabolic panel, liver function tests, and beta-human chorionic gonadotropin BUN: Blood urea nitrogen

Lab	Patient value	Normal value
White blood cells	7.7 x 10^9^/L	4.5-11 x 10^9^/L
Red blood Cells	4.5 million/mm^3^	3.5-5.5 million/mm^3^
Platelets	238,000 /mm^3^	150,000 - 400,000 /mm^3^
Neutrophils	61.0%	54-62%
Eosinophils	2.6%	1-3%
Lymphocytes	27.9%	25-33%
BUN	13 mg/dl	7-18 mg/dl
Creatinine	0.7 mg/dl	0.6-1.2 mg/dl
Hemoglobin	13.0 g/dl	12-16 g/dl
Hematocrit	40.8%	36 - 46%
Alanine aminotransferase (ALT)	27 U/mL	10-40 U/L
Aspartate aminotransferase (AST)	18 U/L	12-38 U/L
Alkaline phosphatase	57 U/L	25-100 U/L
Albumin	4.4 g/dl	3.5-5.5 g/dl
Bilirubin, Total	0.8 mg/dl	0.1-1.0 mg/dl
Beta-human chorionic gonadotropin (β-hCG)	<3.4 IU/L	<5 IU/L for non-pregnant

**Figure 3:**
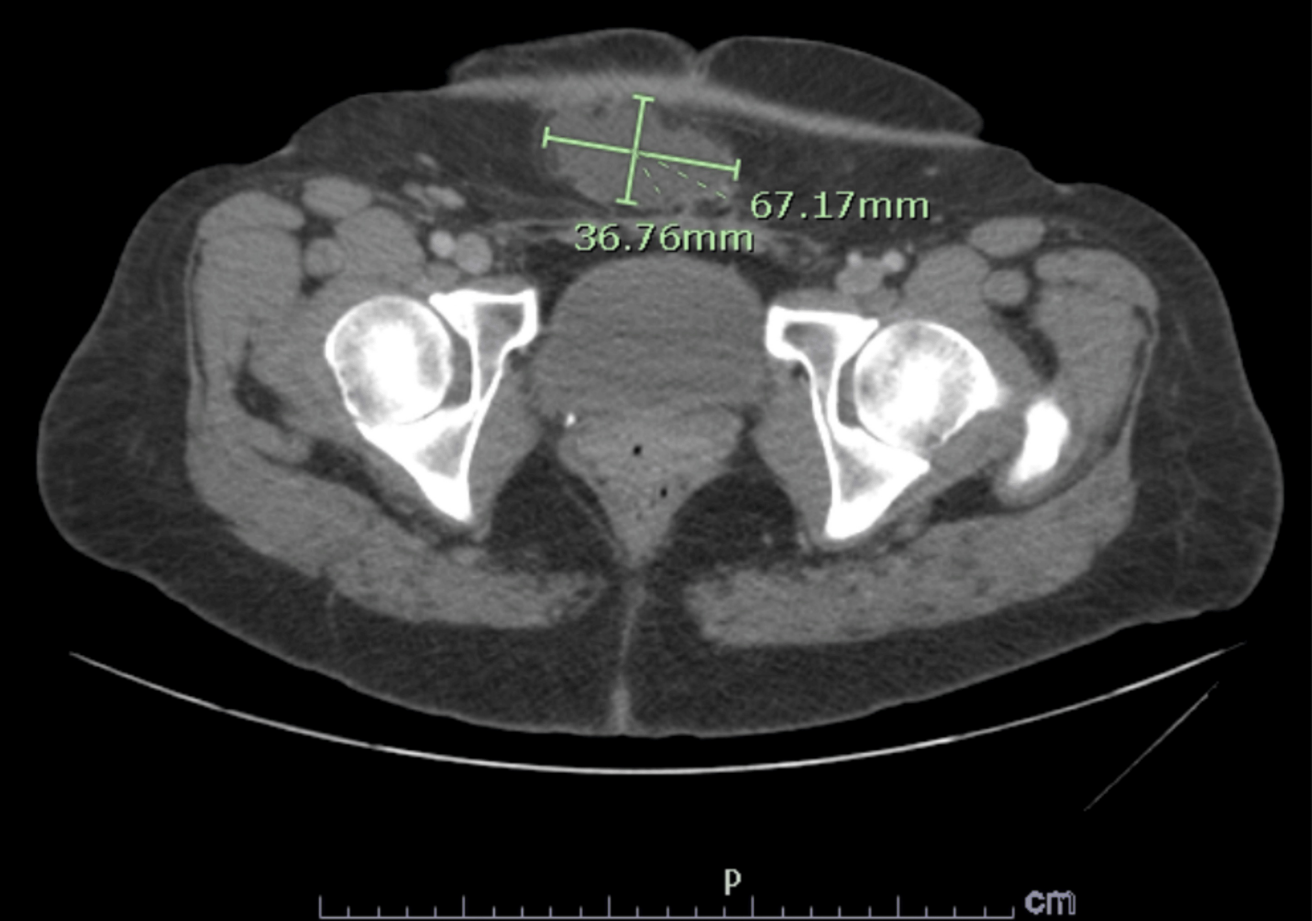
Axial CT abdomen view of the enlarging cystic mass that is now 6.7cm x 3.6cm. The nodule has irregular margins with mild spiculations and is directly adherent to the fibrotic, Pfannenstiel scar tissue.

**Figure 4:**
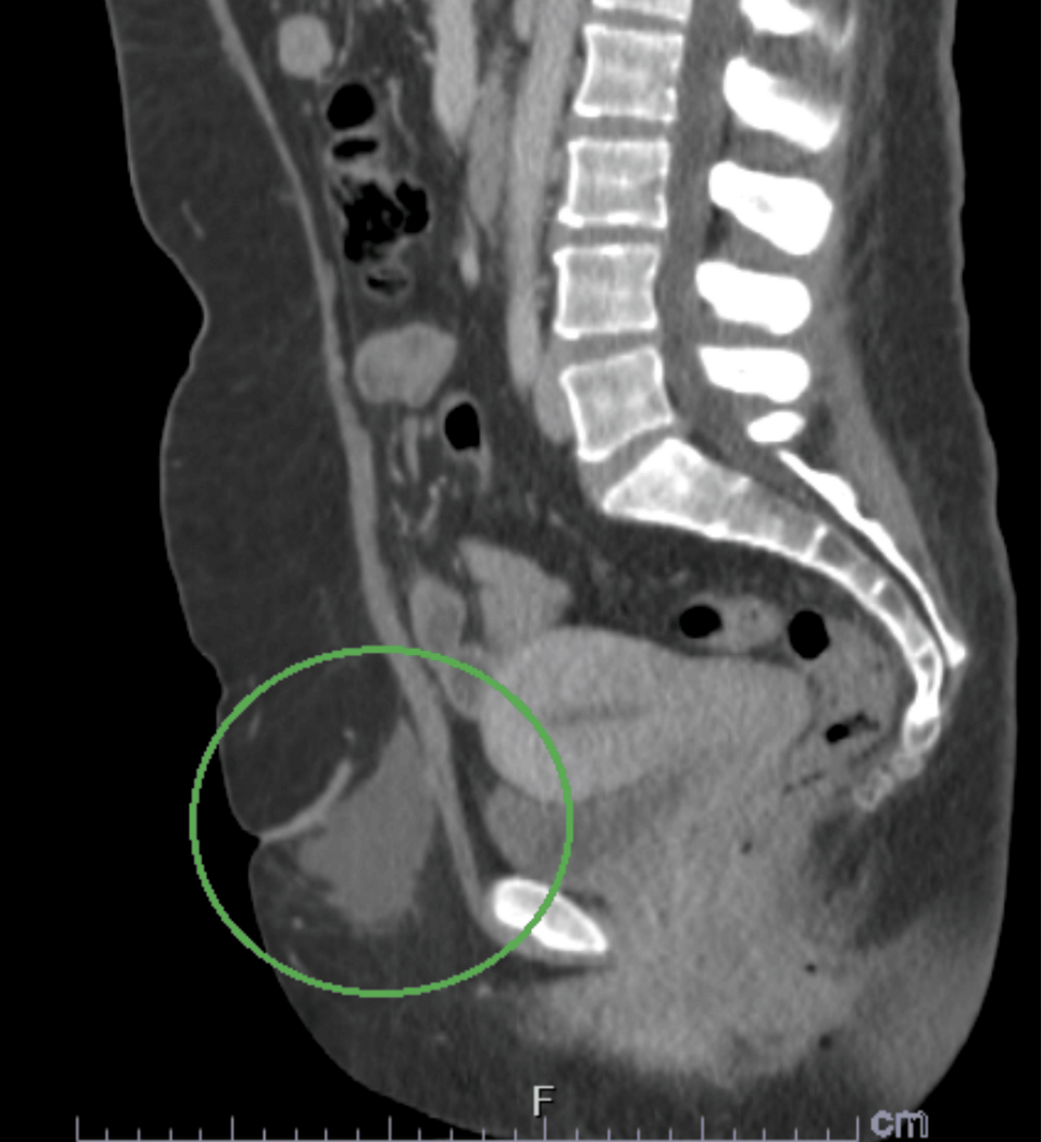
Sagittal CT abdomen view of the enlarging cystic mass that is now 6.7cm x 3.6cm. The nodule has irregular margins with mild spiculations and is directly adherent to the fibrotic, Pfannenstiel scar tissue.

On physical exam, the lower mid-abdomen was tender upon palpation near the site of the prior cesarean scar tissue. An abdominal CT was ordered to assess for a significant tumor mass-effect or possible deep abscess. Imaging revealed a cystic, soft-tissue mass in the subcutaneous tissue just posterior and deep to the abdominal muscles (Figures 3, 4).

The following day, the patient was admitted to the inpatient floor, Interventional radiology was consulted due to the abnormal CT findings on the day prior. Although there was high clinical suspicion of incisional endometriosis, hormonal contraception treatment, usually the first line for symptomatic relief of endometriosis, was deferred for multiple reasons. Open laparotomy, when compared to both laparoscopic surgery and contraception, was preferred due to the large size and well-localized seeding into the superficial, subcutaneous tissue. The patient's above-average BMI and large size of the endometrioma in particular made it difficult for an ultrasonographic ablation approach. As a result, the Interventional Radiologist discussed with General Surgery who then opted for open surgical intervention. This decision was deemed more appropriate than symptomatic or conservative management (combined contraceptives) due to her ongoing attempts to conceive and her history of migraines. For similar reasons, marginal excision of the endometrioma was also chosen over definitive hysterectomy to preserve future fertility. These decisions were reinforced and supported by current radiologic and obstetric literature, discussed below.

During the excisional biopsy, a standard cautery-dissection 15-blade was used for the well-defined, palpable mass. No significant spillage or bleeding occurred, and complete, circumferential excision was performed to separate the mass from the underlying fat and muscle. Samples of the resected mass were then sent to surgical pathology which described scanty smooth muscle stroma and active glandular tissue definitive for typical endometriosis. The patient was then discharged with minimal complications. As part of the post-operative care, the patient was given Ketorolac and other non-steroidal anti-inflammatory drugs (NSAIDs) for pain control and discharged later that week. The patient was informed of possible recurrence and standard post-operative suppressive hormonal therapy but remained firm on her desire for immediate pregnancy. She was instructed to avoid sexual intercourse but was assured that her fertility would return to normal function within the next two months. Her two-week follow-up was without complications, and she is scheduled to follow up with her OB/GYN later this year and has not since reported any abdominal symptoms or pain.

## Discussion

AWE includes cesarean scar endometriosis (CSE), a type of incisional endometriosis. Incisional endometriosis also has occurred during other gynecologic procedures, such as hysterectomies, episiotomies, salpingostomies, and laparoscopic surgeries [5]. CSE has a reported incidence of 0.03-0.45% and is best explained by the direct inoculation theory, where endometrial tissue is transferred directly into the incision during surgery [9]. The hypothesized mechanism has also been proposed to include irritative and vasogenic changes following delivery [6]. Symptoms of CSE include a palpable abdominal mass, dysmenorrhea, and cyclical pain. The duration between cesarean section and the onset of symptoms was previously found to be a mean of 28 months with a standard deviation of 25 months [9]. Our patient experienced an onset of symptoms several months postpartum, which is less than the mean onset of symptoms reported in some studies [1,8,10]. One possibility is the association of obesity with more advanced endometriosis due to a strong association from elevated estrogen levels in excessive adipose tissue as endometriosis is an estrogen-dependent disease [10]. It is highly likely that the patient's clinical obesity (BMI of 34) contributed to her rapid mass progression and earlier onset of symptoms. Counseling the patient about lifestyle modifications for weight loss may have reduced progression of the disease. 

One important shift in recent years is the increase in the incidence of AWE seen due to higher rates of elective cesarean sections and gynecological laparoscopic surgeries in recent decades [4,5,7]. There is ongoing debate in the literature and it is well documented that this upward trend contributes to current standards and indications for elective cesarean treatment. The Pfannenstiel incision, the preferred approach to cesarean section, is a low, transverse incision located approximately 2-3 cm above the symphysis pubis. It has succeeded as the new standard over vertical incision due to cosmetic reasons, reduced postoperative pain, reduced risk of incisional hernia, and easier repair [11]. Alternatively, the Pfannenstiel incision has wider surgical dissection planes which may be more easily inoculated with endometrial tissue [12]. Many methods have been proposed to reduce CSE in Pfannenstiel incisions, including thorough irrigation and careful instrumentation [9]. Despite recommendations, it is possible that these methods were not completely utilized in our patient in the past, resulting in a higher risk and subsequent CSE in our patient. In addition to a cesarean section, other risk factors for incisional endometriosis include heavy menstrual flow and alcohol consumption [13] which our patient did not report. As the prior cesarean section was a major factor and due to the location of the endometrioma, future consideration of proper surgical techniques may reduce the seeding of endometrial tissue into the incision and CSE risk. This may not be as concerning in smaller endometriomas or those treated appropriately with high-intensity focused ultrasound (HIFU) ablation.

Suspected CSE is typically evaluated initially with ultrasound, followed by CT or MRI [5,6]. Due to the patient’s initial presentation of right upper quadrant pain from acute cholecystitis, this mass was incidentally discovered with CT upon surgical workup without a prior ultrasound of the lower abdomen. Official diagnosis can only be made with histopathological findings, by surgical excision of the mass or fine needle aspiration [6]. HIFU and surgical excision have both been shown to be safe and effective treatments for AWE. There are no differences in complication rate, recurrence of AWE, and pain levels on follow-up between the two treatments, although more research is needed comparing the two [14]. As mentioned above, there is a possibility that the size limitation and smaller endometriomas treated with HIFU can reduce some of the complication burden or risk seen with larger, excised endometriomas. Among other benefits, HIFU has been shown to result in shorter hospital stays and reported post-operative pain. Additionally, there is a cosmetic benefit for women who are concerned about residual scars as HIFU does not cause any abdominal incisions [15]. Although the benefits of HIFU are many, one of the biggest shortcomings is size limitations with a resectable mass having a diameter below 3-4 cm as mentioned previously [16]. While HIFU may be appropriate for small-to-moderate-sized masses, this patient’s larger mass exceeding 6cm, leaves surgical excision as a more preferential treatment of choice. Progestins, combined oral contraceptive pills, and NSAIDs are first-line treatments for endometriosis-associated pain and symptomatic management [17]. Hysterectomy is a last-line treatment and is appropriate for patients who do not desire future fertility and have not responded to more conservative management [17]. Our patient also desired immediate, future fertility so contraceptive management or hysterectomy was deferred in this case.

Lastly, it is important to note that endometriosis has been shown to undergo a rare, malignant transformation in as few as 0.7-1.5% of all endometrioma cases, with 79% of these neoplastic transformations occurring in the ovaries [18]. CSE was the most common site for malignant transformation of AWE, with an incidence of 0.3-1%. If left untreated, subsequent malignant transformation archetypes include clear cell cancer followed by endometrioid adenocarcinoma [18]. It is therefore important to be proactive in treating symptomatic endometriosis, especially in atypical locations such as adjacent to the abdominal wall. Some studies report malignant Pfannenstiel endometrioma transformations to have significant mortality, nearly upwards of 43% if left untreated [7].

## Conclusions

One of the goals of our case report is to highlight the presentation of Pfannenstiel endometrioma and decision making that indicates early intervention. Although rare, Pfannenstiel endometrioma should be considered a differential diagnosis for a patient with a previous cesarean section presenting with cyclical abdominal pain and a palpable tenderness along the incision site. CSE may also be asymptomatic, as seen in this patient upon the incidental discovery of the endometrioma. Due to this, shared decision making between the patient and physician is necessary to determine preference for treatment. In this case, the patient was managed conservatively for several months until her symptoms worsened, as surgical management was not performed after an initial incidental finding on CT imaging. While no official guidelines have been established for management of cesarean section endometriosis, it is important to be familiar with conservative and surgical options for patients.

One alternative way to approach CSE is to increase efforts in reducing risk factors for more advanced endometriosis disease progression, such as obesity. As it is well established, contraceptives and NSAIDs may be helpful for symptomatic management. However, removal of the endometrioma, i.e. HIFU or surgical excision are both options for definitive management. Definitive management may not only prevent the common symptoms of chronic pain, discomfort, and dysmenorrhea but also lower the risk of malignant transformation. This should be taken into consideration when deciding to surgically treat a patient with Pfannenstiel endometriosis, however, it is noted that malignant transformation is rare and occurs more frequently in other locations. Importantly, preventing and reducing risk for the development of Pfannenstiel endometriosis starts at the initial cesarean section procedure, by using surgical techniques to minimize seeding into the incision. Lastly, the upward trend of elective cesarean sections poses an additional challenge for radiographers and clinicians toward the already, highly debated management guidelines of atypical endometriomas. By introducing this case, we hope to contribute to additional and to guide clinician decision-making to proceed or withhold interventions on endometriomas, incidental or atypical.
